# A Nano-Based Approach to Deliver *Satureja thymbra* Essential Oil to the Skin: Formulation and Characterization

**DOI:** 10.3390/molecules29051041

**Published:** 2024-02-28

**Authors:** Simone Pani, Carla Caddeo, Cinzia Sanna, Francesca Pintus, Sonia Floris, Ramon Pons, Aurélien Dupont, Carlo Ignazio Giovanni Tuberoso

**Affiliations:** 1 Department of Scienze della Vita e dell’Ambiente, University of Cagliari, S.P. Monserrato-Sestu Km 0.700, 09042 Monserrato, Italy; simone.pani@unica.it (S.P.); fpintus@unica.it (F.P.); s.floris@unica.it (S.F.); tuberoso@unica.it (C.I.G.T.); 2 Department of Life and Environmental Sciences, University of Cagliari, Via S. Ignazio da Laconi 13, 09123 Cagliari, Italy; cinziasanna@unica.it; 3 Department of Surfactants and Nanobiotechnology, Institute for Advanced Chemistry of Catalonia (IQAC-CSIC), c/Jordi Girona, 18–26, 08034 Barcelona, Spain; ramon.pons@iqac.csic.es; 4 Biosit—UMS 3480, US_S 018, University of Rennes, F-35000 Rennes, France; aurelien.dupont@univ-rennes.fr

**Keywords:** *Satureja thymbra*, essential oil, phytochemical profile, antioxidant, phospholipid vesicles, skin delivery

## Abstract

Essential oils are well known for their biological properties, making them useful for the treatment of various diseases. However, because of their poor stability and high volatility, their potential cannot be fully exploited. The use of nanoformulations to deliver essential oils can solve these critical issues and amplify their biological activities. We characterized an essential oil from *Satureja thymbra* via GC–MS and HPLC–DAD to provide qualitative and quantitative data. The essential oil was formulated in phospholipid vesicles which were characterized for size, surface charge, and storage stability. The entrapment efficiency was evaluated as the quantification of the major monoterpenoid phenols via HPLC–DAD. The morphological characterization of the vesicles was carried out via cryo-TEM and SAXS analyses. The essential oil’s antioxidant potential was assayed via two colorimetric tests (DPPH^•^ and FRAP) and its cytocompatibility was evaluated in HaCaT skin cell cultures. The results showed that the nanoformulations developed for the loading of *S. thymbra* essential oil were below 100 nm in size, predominantly unilamellar, stable in storage, and had high entrapment efficiencies. The vesicles also displayed antioxidant properties and high cytocompatibility. These promising findings pave the way for further investigation of the therapeutic potential of *S. thymbra* nanoformulations upon skin application.

## 1. Introduction

*Satureja* (savory) is a genus of the Lamiaceae family and includes aromatic plants native to southern and southeastern Europe, North Africa, Central Asia, and the Middle East. Some *Satureja* species are cultivated all over the world as spices or flavoring agents, owing to their intense aroma. They are also widely used for medical purposes (e.g., to treat muscle pains, stomach and intestinal disorders, infectious diseases, and as tonics, relievers, and diuretics) [1,2,3]. Most are rich in essential oils, as are the majority of Lamiaceae species [4]. Essential oils are known for their biological properties, especially their antimicrobial activity. In tests against common pathogens, they have shown high antimicrobial activity that supports their use in the treatment of wounds and disorders of the oral cavity [4].

*Satureja thymbra* L. is a perennial shrub that grows in the East Mediterranean basin from Turkey and Greece to Sardinia (Italy), the latter representing the western limit of its distribution range. It is known for its ease of cultivation and sweetness, which makes it a common flavoring agent in pharmaceutical products and foods. *S. thymbra* is also used as a home remedy due to its antiseptic and gastrosedative properties [5]. In Sardinia, *S. thymbra* has been traditionally used as a digestive, antispasmodic, antidiarrheic, diuretic and antiseptic, and to promote wound healing [6]. Moreover, it has been used to treat malaria, and this traditional practice has been supported by a study of the antiplasmodial activity of *S. thymbra* essential oil [7].

Essential oils are complex mixtures, and their biological activities vary in accordance with their components, which can act synergistically to confer broader and stronger activity [8,9]. Previous investigations of the chemical composition of *S. thymbra* essential oil have revealed the phenols carvacrol and thymol to be its major components, contributing to its antimicrobial and antifungal properties [10,11]. Due to these active components, *S. thymbra* essential oil has very strong potential for pharmaceutical applications. However, this potential is limited by the essential oil’s low stability in direct exposure to light, heat, and humidity, as well as its high volatility, which reduces its therapeutic applicability [12].

Nanocarriers, such as phospholipid vesicles, can effectively overcome these limitations, representing a leading strategy to formulate essential oils. With this approach, the essential oil is protected from degradation and delivered in an aqueous formulation, thus making it more feasible for application in the medical field. In addition to enhanced bioavailability, phospholipid vesicles also ensure controlled release to increase efficiency and reduce toxicity [13].

**Figure 1:**
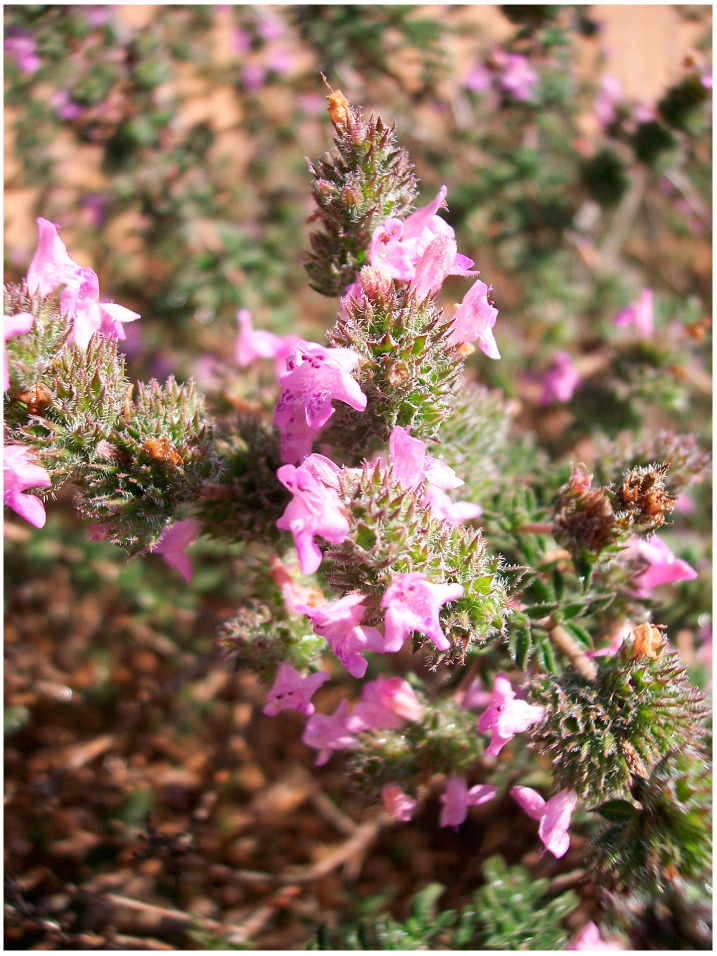
Flowering aerial parts of *Satureja thymbra* L.

In this study, an essential oil produced from *S. thymbra* flowering aerial parts (Figure 1) was characterized via GC–MS to gain qualitative and abundance data on the volatile compounds, while the phenolic composition was determined via HPLC–DAD. The novelty of this study lies in exploring the possibility to produce, via a facile and efficient method, a topical nano-based formulation of the *S. thymbra* essential oil, with a special focus on its stability, retained bioactivity, and safety. Phospholipid vesicles, namely liposomes and Et-PEVs (liposomes modified with a skin penetration enhancer, i.e., ethanol) were produced and characterized by light scattering for size, charge, and storage stability. The oil’s entrapment efficiency was determined as the quantification of the major phenolic compounds via HPLC–DAD. The morphology was evaluated with cryo-TEM and SAXS. The antioxidant potential of the nanoformulations was determined colorimetrically and their safety was assessed in skin cell cultures.

## 2. Results

### 2.1. Quali-Quantitative Determination of Volatile Compounds in S. thymbra Essential Oil

**Figure 2:**
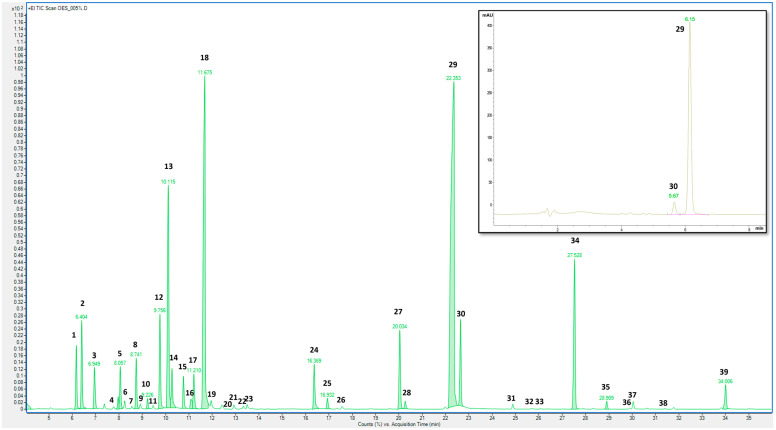
GC–MS chromatogram of *S. thymbra* essential oil. In the upper right corner is the HPLC–DAD chromatogram at 210 nm of *S. thymbra* essential oil. Peak identification is given in Table 1.

The *S. thymbra* essential oil was analyzed via GC–MS to gain qualitative and abundance data for the volatile compounds. The GC–MS profile (Figure 1) showed the presence of 39 compounds, with thymol (peak 29), γ-terpinene (peak 18), and p-cymene (peak 13) being the most abundant (Table 1), confirming previous observations of *S. thymbra* essential oil from Sardinia [7,9,11]. A validated HPLC–DAD method was applied to quantify two monoterpenoid phenols, carvacrol and thymol, in the raw essential oil and in the nanoformulations. The chromatogram at 210 nm is presented in Figure 2, which shows a higher amount of thymol than carvacrol (470.25 and 18.10 mg/L, respectively). This aligns with the GC data showing 23.88% of thymol and 7.08% of carvacrol (Table 1).

### 2.2. Characterization of Liposomes and Et-PEVs

The *S. thymbra* liposomes and Et-PEVs were characterized in terms of size, homogeneity, and charge and compared with the empty liposomes and empty Et-PEVs in order to evaluate possible modifications to the main vesicles’ characteristics due to the presence of the essential oil (Table 2). For the empty liposomes, the mean diameter was 96 nm. It decreased upon incorporation of the essential oil (86 nm; *p* < 0.001), which also improved the homogeneity of the system, as the PI decreased significantly from 0.33 to 0.20, a value indicative of a very good size homogeneity of these vesicles. The same pattern was observed for the Et-PEVs: the empty Et-PEVs were significantly larger than that of the *S. thymbra* Et-PEVs (107 nm vs. 79 nm) and the PI decreased significantly from 0.37 to 0.24 (Table 2). The zeta potential values became more negative in the presence of the essential oil for both liposomes and Et-PEVs (from −12 to −18 mV; Table 2). The addition of ethanol in empty Et-PEVs induced an enlargement of the vesicles in comparison with empty liposomes (107 nm vs. 96 nm; Table 2), while the opposite effect was observed when the essential oil was also present (86 nm for *S. thymbra* liposomes and 79 nm for *S. thymbra* Et-PEVs; Table 2). The entrapment efficiency of the nanoformulations was evaluated as the quantification of two monoterpenoid phenols identified in the *S. thymbra* essential oil. Both liposomes and Et-PEVs showed very high entrapment efficiencies, especially the Et-PEVs: 87 and 90% vs. 95 and 96% for carvacrol and thymol (detected at *tr* 5.66 and 6.15 min by HPLC–DAD; Figure 1), respectively (Table 2).

**Figure 3:**
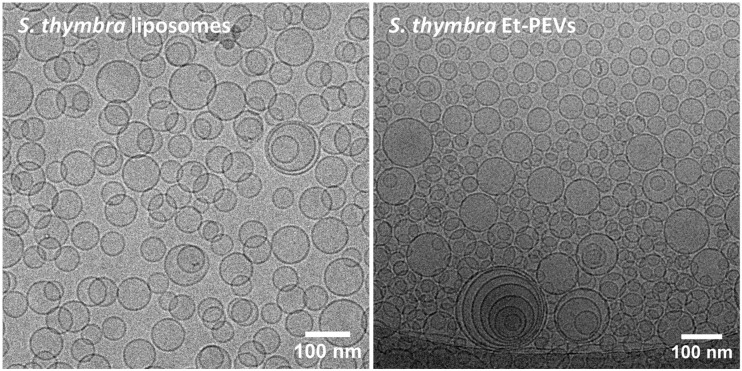
Cryo-TEM micrographs of *S. thymbra* liposomes and Et-PEVs.

The storage stability of the liposomes and Et-PEVs was evaluated by monthly measurements of their mean diameter, polydispersity index, and zeta potential. No evidence of significant alterations was detected for *S. thymbra* vesicles: after 90 days at 4 °C, the mean diameter was around 85 nm, the PI was below 0.3, and the zeta potential was around −18 mV. On the contrary, the empty vesicles displayed evidence of instability, as the mean diameter increased to approximately 120 nm, as well as the polydispersity (PI 0.49) and the zeta potential (−20 mV). This confirms that the essential oil played a key part in the formation of smaller and more homogeneous vesicles, which remained basically unaltered throughout the period of time monitored. In addition, the nanoformulations were stored at room temperature. Only the *S. thymbra* Et-PEVs maintained their physico-chemical features for 90 days, which points to a contribution by ethanol to the vesicles’ stability. The morphological evaluation performed by cryo-TEM indicated that the *S. thymbra* liposomes were characterized predominantly by a unilamellar structure and an average size below 100 nm (Figure 3). The same size was observed in *S. thymbra* Et-PEVs, which were unilamellar with a more relevant fraction of oligolamellar vesicles (Figure 3).

**Figure 4:**
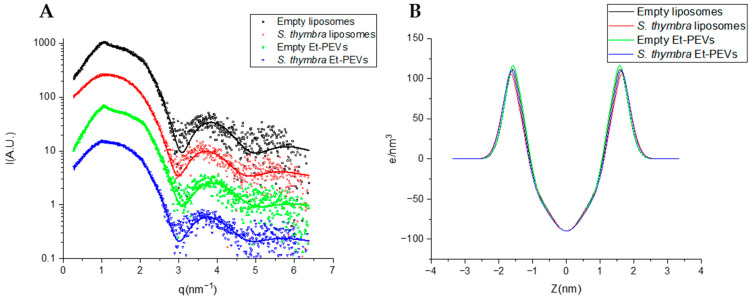
(**A**) SAXS curves of empty liposomes, *S. thymbra* liposomes, empty Et-PEVs, and *S. thymbra* Et-PEVs. The fits of the bilayer model are shown as lines. (**B**) Electron density profiles used for the fitting of empty liposomes, *S. thymbra* liposomes, empty Et-PEVs, and *S. thymbra* Et-PEVs.

SAXS analyses were performed to deeper characterize the structure of the vesicles. The SAXS curves, which are displayed in Figure 4, along with the fits of the lamellar model, were characteristic of bilayered structures. Table 3 shows the main parameters obtained from the fits. Reduced chi-squared values were below 2 (*χ*^2^ = 1.3–1.8; Table 3), which is indicative of a very good fit of the applied model to the SAXS data. The bilayer electronic distribution of the four samples were very similar (Figure 4B), which is due to their similar electron densities, as indicated by the headgroup contrast electron density values (*ρ_H_*; Table 3). Based on the shape of the SAXS curves, both empty vesicles show clear evidence of multilayered structures (Figure 4A), more precisely, oligolamellar, and in a small proportion, about 12–14% (Table 3). In the empty liposomes, the number of correlated bilayers *N_C_* was 3.5, at a repetition distance d of 58 Å, and with a Caillé parameter *η*_1_ = 0.18 (a value indicative of flexible bilayers). Similar values were measured for the empty Et-PEVs (Table 3). These parameters could not be measured for the *S. thymbra* liposomes (Table 3), which points to a predominance of unilamellar structures. On the other hand, in *S. thymbra* Et-PEVs there still was a faint presence of multilayered structures (~6%; Table 3). Hence, the incorporation of the essential oil reduced the lamellarity, especially in the liposomes. This is congruent with the increase in charge detected by the zeta potential measurements (Table 3). Moreover, the distance between the polar heads and the bilayer center (*Z_H_*) increased slightly with the incorporation of the essential oil in both liposomes and Et-PEVs (Table 3). The polar head (*σ_H_*) and the methyl (*σ_C_*) amplitude slightly increased when the essential oil was incorporated in the liposomes and decreased in the *S. thymbra* Et-PEVs (Figure 4). Nevertheless, the differences were small.

### 2.3. Antioxidant Activity

The antioxidant activity of the *S. thymbra* essential oil was estimated based on its radical scavenging and ferric reducing abilities. Based on the DPPH^•^ test, the antioxidant activity of the *S. thymbra* liposomes and Et-PEVs was significantly higher (84 and 85%, corresponding to 164 and 171 μg TE/mL, respectively; Table 4) than that of the solution (65%, corresponding to 124 μg TE/mL; Table 4). Due to the presence of phosphatidylcholine and to the high volume (40 μL) of vesicle dispersion used for the test, the empty liposomes and empty Et-PEVs exhibited an antioxidant activity level similar to that of *S. thymbra* solution (60% and 69%, corresponding to 116 μg and 142 μg TE/mL, respectively; Table 4). Therefore, the higher activity of the *S. thymbra* nanoformulations was due to a combined effect of the essential oil and the phospholipid. Moreover, these data prove that the antioxidant activity of the essential oil was not altered during the vesicles’ preparation, but rather potentiated by the nanoformulation. The results of the FRAP assay, on the other hand, indicated that the *S. thymbra* solution and the *S. thymbra* liposomes/Et-PEVs had a striking reducing power, which was ten times higher than that of the empty vesicles (12 vs. 1.2 mg FE/mL; Table 4).

### 2.4. Viability of Skin Cells

**Figure 5:**
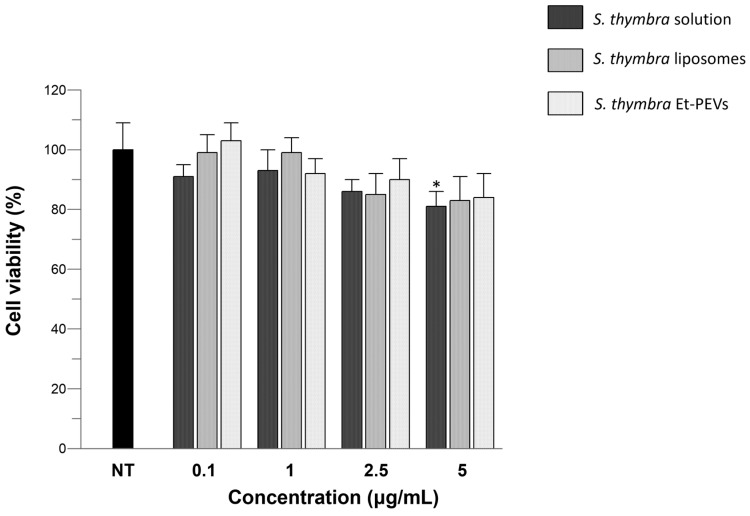
Effects of the *S. thymbra* ethanol solution and *S. thymbra* liposomes/Et-PEVs on the viability of HaCaT cells incubated for 24 h with different concentrations (0.1–5 μg/mL). *: *p* < 0.05 vs. non-treated control cells (NT).

The effects of the *S. thymbra* solution and *S. thymbra* liposomes/Et-PEVs on the viability of keratinocytes were examined. The results indicated that, after 24 h of exposure to *S. thymbra* samples, none of them were cytotoxic. Only a slight, still statistically significant, decrease in viability was detected in the cells exposed to the higher concentration (5 μg/mL) of *S. thymbra* solution, reaching a lowermost value of 81% (Figure 5). At the same concentration, no statistically significant difference was detected in the cells incubated with the *S. thymbra* liposomes/Et-PEVs (Figure 5).

## 3. Discussion

Essential oils from many plant species have become popular in recent years. *S. thymbra* is well known for its essential oil, which is rich in monoterpenes. In accordance with Glamoclija et al. [14], we found that thymol, γ-terpinene, *p*-cymene, and carvacrol were the most abundant compounds in this essential oil. The percentage values (Table 1) of thymol (23.88%), γ-terpinene (20.38%), and *p*-cymene (7.27%) found in our essential oil were similar to those found by Capone et al. [9] (22.16%, 29.39%, and 8.76%, respectively), while Piras et al. [11] and Dell’Agli et al. [7] detected higher levels of thymol (57.3% and 49.60%, respectively). In contrast to our results, however, Piras et al. [11] detected lower amounts of γ-terpinene (9.8%). These differences in the chemical composition of the essential oils obtained from Sardinian *S. thymbra* samples could be attributable to the different distillation methods adopted (steam distillation, hydrodistillation, or supercritical fluid extraction) and the harvesting period. In particular, Dell’Agli et al. [7] documented high variability in the essential oil of *S. thymbra* collected in three different periods, highlighting increasing amounts of thymol before, during, and after flowering (36.0%, 52.6%, and 57.1%, respectively). From these same periods, Dell’Agli et al. [7] found decreasing percentages of γ-terpinene (27.0%, 18.2%, and 11.8%, respectively). Another abundant compound detected in our *S. thymbra* essential oil was carvacrol (7.08%; Table 1). Our essential oil, as well as the others obtained from Sardinian samples, was characterized by a very low amount of carvacrol compared to Greek essential oils, confirming the existence of two different carvacrol and thymol chemotypes, as widely reported in the literature [3,15,16]. Our results are also in accordance with previous findings on the relationship between altitude and *S. thymbra* essential oil’s chemical composition [17,18]. The total yield and amount of thymol were negatively correlated with altitude, while better yields of carvacrol were correlated with higher altitudes. Thymol and carvacrol are considered strong antioxidant agents, as proven by studies that investigated not only the whole oil, but also the isolated phenols [19,20]. Their strong antioxidant power was proved by Giweli et al. [21] using the DPPH^•^ radical scavenging test. In another study, their antioxidant activity was demonstrated by β-carotene-linoleic acid, DPPH^•^, ABTS^•^, and CUPRAC assays [22]. These findings support the use of the *S. thymbra* essential oil to protect against oxidative stress. The antioxidant activity of our essential oil was investigated with the DPPH^•^ and FRAP tests. The latter showed striking reducing power, while the former showed moderate scavenging activity. These differences suggest that the essential oil exerts its antioxidant activity by reducing ferrous ions rather than free radicals. The DPPH^•^ scavenging activity of the essential oil incorporated into liposomes and Et-PEVs was significantly higher, thanks to a combined effect of the essential oil and the vesicles’ phospholipid. This demonstrates that the essential oil’s bioactivity was potentiated by the nanoformulation. Both the liposomes and Et-PEVs were small in size and stable in storage. The mean diameter of the vesicles decreased upon incorporation of the essential oil, which also improved the homogeneity of the system. In another study, a *S. thymbra* essential oil was incorporated into two different types of vesicles, propylene glycol-nanovesicles and glycerosomes, at the same concentration used in our study (10 mg/mL) [23]. Unlike our study, those vesicle formulations were proposed as food preservatives, owing to their antimicrobial activity against food-borne bacteria and fungi [23]. In agreement with our study, Vanti et al. [23] also demonstrated the safety of the vesicle formulations in a cellular system. Overall, these findings point to a broad range of applications for *S. thymbra* essential oil, which encourages further exploration of its nanoformulations for healthcare.

## 4. Materials and Methods

### 4.1. Materials

The standards for thymol, carvacrol, and terpenes were from Extrasynthese (Genay Cedex, France). The LC–MS-grade acetonitrile, methanol, ethanol 96%, 85% phosphoric acid, Trolox (6-hydroxy-2,5,7,8-tetramethylchroman-2-carboxylic acid), ferrous sulfate heptahydrate, and all other products, if not otherwise specified, were from Sigma-Aldrich/Merck (Milan, Italy). The Phospholipon 90G (soy phosphatidylcholine content ≥ 94%) was from Lipoid GmbH (Ludwigshafen, Germany).

### 4.2. Plant Material and Preparation of the Essential Oil

The flowering aerial parts of *Satureja thymbra* were harvested during the blooming period (June 2021) in Colle San Michele (Cagliari, Italy, 39°14′45.3′′ N 9°06′37.6′′ E). The species was authenticated by Cinzia Sanna and an exsiccatum was deposited at the Herbarium CAG of the University of Cagliari with the voucher CAG1079/v1. The fresh plant material (980 g) was subjected to steam distillation to isolate the volatile components, as described by Dell’Agli et al. [7] with slight modifications. After 3 h, 8 mL of essential oil was obtained. The essential oil was left to stabilize for 1 h with anhydrous Na_2_SO_4_ to eliminate any residual water, then it was filtered and kept in the dark until use.

### 4.3. GC–MS Analysis

The essential oil’s volatile compounds profile was determined via GC–MS using an 8890-gas chromatograph (Agilent Technologies, Palo Alto, CA, USA) equipped with an Agilent 7693A autosampler and fitted with an Agilent 5977B single quadrupole mass detector. Separations were performed on an Agilent HP-5MS capillary column (30 m × 0.25 mm, 0.25 μm). The oven temperature ramped up from 50 °C for 1 min to 220 °C at 3 °C/min and was held isothermal for 13 min. Helium (1 mL/min) was used as the carrier gas. The injected volume was 1 μL. The injector operated in splitless mode at 250 °C. The MS transfer line, source, and quadrupole were set at 280 °C, 280 °C, and 150 °C, respectively. The quadrupole mass spectrometer was operated at an electron ionization energy of 70 eV, scanning in the *m*/*z* mass range of 50–650 average mass unit (amu). Acquisition and elaboration of chromatograms was performed with the qualitative and quantitative tools of an Agilent MassHunter Workstation v. 10.2. Peaks were assigned by comparing the retention indices (RIs) relative to C_8_–C_24_ *n*-alkanes with those of standards and/or those found in the literature [24,25], as well as by comparing the mass spectra with the [24] mass spectral database and the Wiley 275 MS library (New York, NY, USA). The percentage composition of the essential oil, which was calculated as the mean of three analyses, was computed from the GC peak areas using the normalization method without correction factors. The essential oil was diluted 0.5:100 *v*/*v* with *n*-hexane before analysis.

### 4.4. HPLC–DAD Analysis

The essential oil’s monoterpenoid phenol profile was determined via HPLC–DAD as described by [26] using an Agilent 1260 Infinity II HPLC system (Cernusco sul Naviglio, Milan, Italy) fitted with a G7111A pump, a G7129A autosampler, a G7116A thermostatted column compartment (30 °C), and a G4212B photodiode array detector. Separations were performed on a Phenomenex Kinetex EVO C18 column (150 mm × 4.60 mm, 2.6 μm; Casalecchio di Reno, Bologna, Italy) using a mobile phase of 0.22 M phosphoric acid:acetonitrile (60:40 *v*/*v*) at an 0.8 mL/min flow rate. The injected volume was 10 μL. The chromatograms and spectra within the wavelength range of 200–600 nm were processed with an OpenLab data system v. 2.51 (Agilent Technologies). Standards (thymol and carvacrol) stock solutions were prepared in methanol and further dilutions were made with 0.22 M phosphoric acid [26]. The standard calibration curves were built in a 1–200 mg/L range using the external standard method, with a coefficient of determination (*r^2^*) ≥ 0.9993 for both carvacrol and thymol. The LODs and LOQs were calculated according to the equation LOD = 3.3 *r*/*S* and LOQ = 10 *r*/*S*, respectively (where *r* = standard deviation of the blank and *S* = slope of the calibration curve) [26], resulting in LOD ≥ 0.12 mg/L and LOQ ≥ 0.35 mg/L for both carvacrol and thymol. The essential oil was dissolved in MeOH (1:10 *v*/*v*) and then diluted (1:10 *v*/*v*) in an 80:20 *v*/*v* MeOH:0.22 M H_3_PO_4_ mixture. The nanoformulations were first diluted with methanol (1:50 *v*/*v*) and then with an 80:20 *v*/*v* MeOH:0.22 M H_3_PO_4_ mixture (1:10 *v*/*v*) before injection.

### 4.5. Preparation of the Phospholipid Vesicles

Liposomes were prepared by dispersing the phospholipid in water (90 mg/mL), with or without the *S. thymbra* essential oil (10 mg/mL). Et-PEVs were prepared by adding ethanol 96% (50 μL/mL) to the phospholipid and water, with or without the essential oil (10 mg/mL). Both the dispersions were sonicated for 5 sec ON/2 sec OFF for 9 cycles using an ultrasonic disintegrator (Soniprep 150 plus, MSE Crowley, London, UK).

### 4.6. Characterization of the Phospholipid Vesicles

The average diameter, polydispersity index, and zeta potential of the liposomes and Et-PEVs were measured by dynamic and electrophoretic light scattering [27] using a Zetasizer nano-ZS (Malvern Panalytical, Worcestershire, UK). These parameters were analyzed over 90 days to determine the storage stability (at 4 and 25 °C) of the vesicles. The *S. thymbra* liposomes and Et-PEVs were dialyzed against water to remove non-incorporated essential oil components [28]. Both non-dialyzed and dialyzed samples were diluted with methanol (1:50 *v*/*v*) and analyzed by HPLC–DAD (see Section 4.4). The amounts of the monoterpenoid phenols found in dialyzed and non-dialyzed samples were used to calculate the entrapment efficiency of the vesicles. The morphology of the *S. thymbra* liposomes and Et-PEVs was investigated via cryogenic Transmission Electron Microscopy. The samples (3 μL) were deposited onto glow-discharged grids, then blotted and vitrified by rapid freezing into ethane. The vitrification process was carried out using an automatic plunge freezer (EM GP, Leica Microsystems Inc., Deerfield, IL, USA) under controlled humidity and temperature. The samples were observed on a 200 kV electron microscope (Tecnai G2 T20 Sphera, FEI Company/ThermoFisher Scientific, Waltham, MA, USA) equipped with a 4 × 4 k CCD camera (TemCam-XF416, TVIPS GmbH, Gilching, Germany). Micrographs were acquired under low electron dose conditions using the camera in binning mode 1 and at a 25,000× nominal magnification.

### 4.7. Small-Angle X-ray Scattering Analysis

Small-Angle X-ray Scattering (SAXS) analyses were performed to further characterize the vesicles. A description of the equipment and the experimental conditions is reported in [27]. The SAXS curves were recorded every 20 min for 2 h to check sample stability, then summed up (the background was subtracted), fitted using a home-made procedure based on a Gaussian description of the bilayers and a Levenberg–Marquardt minimization scheme [29].

### 4.8. Antioxidant Activity: DPPH^•^ and FRAP Assays

The DPPH^•^ (1,1-diphenyl-2-picrylhydrazyl) assay estimates the antioxidant power of a sample as a function of its radical scavenging activity. The DPPH^•^ is reduced by accepting a hydrogen atom or an electron from antioxidant species: during this process, the color of the DPPH^•^ solution changes from an intense purple to a pale yellow. The decrease in absorbance is proportional to the discoloration of the solution, which in turn is proportional to the antioxidant power of the sample. Forty µL of the samples (i.e., *S. thymbra* in ethanol or in liposomes/Et-PEVs; 10 mg/mL) was added to a DPPH^•^ methanolic solution (25 μm; 2 mL) and incubated for 30 min (in the dark, room temperature); the absorbance was read at 517 nm [28]. The antioxidant activity (AA) of the samples was calculated as follows:

AA (%) = ((A_DPPH_ − A_sample_)/A_DPPH_) × 100

The results were also expressed as µg of Trolox Equivalents (TE)/mL solution, which were calculated by using a calibration curve for Trolox in a 12.5–200 µg/mL range. The antioxidant activity of *S. thymbra* essential oil was also tested with the FRAP (ferric ion reducing antioxidant power) assay. This is based on the reduction of the ferric-tripyridyltriazine complex to an intense, blue-colored ferrous-tripyridyltriazine complex, which causes an increase in absorbance that is proportional to the antioxidant power of a sample. Two µL of the samples (i.e., *S. thymbra* in ethanol or in liposomes/Et-PEVs; 10 mg/mL) was added to the FRAP reagent (2 mL; pH 3.6) and incubated for 4 min (in the dark, room temperature); the absorbance was read at 593 nm [28]. The results, expressed as mg of ferrous equivalents (FE)/mL solution, were calculated by using a calibration curve for ferrous sulfate in a 0.195–12.65 mg/mL range.

### 4.9. Viability of Skin Cells

Human skin keratinocytes (HaCaT; CLS–Cell Lines Service, Eppelheim, Germany) were cultured under standard conditions as previously described [28]. Cell viability was determined by the MTT assay. In short, the cells (10,000 cells/well in 96-well plates) were exposed to *S. thymbra* ethanol solution or *S. thymbra* liposomes/Et-PEVs. The *S. thymbra* samples (initial concentration: 10 mg/mL) were diluted to achieve the following final concentrations: 0.1, 1, 2.5, and 5 µg/mL. After 24 h of exposure, an MTT solution (0.5 mg/mL) was added to the wells, and after 3 h, DMSO was used to dissolve the formed formazan crystals. The absorbance was read at 590 nm.

### 4.10. Statistical Analysis

Results are presented as mean values ± standard deviations (SD). The Student’s *t* test was performed for pairwise comparisons. For cell viability data, one-way ANOVA and Tukey’s post hoc test were applied for comparison of groups using GraphPad Prism software v. 8 (San Diego, CA, USA). Differences were considered significant for *p* < 0.05.

## 5. Conclusions

*S. thymbra* essential oil was formulated in phospholipid vesicles to solve typical issues associated with essential oils (i.e., poor stability and high volatility) and possibly to amplify their biological activities. We characterized the vesicles for key parameters that affect their functionality, such as size, entrapment efficiency, and physical stability. The results showed that *S. thymbra* vesicles were around 80 nm, had uni/oligolamellar structures, entrapped over 80% of the main monoterpenoid phenols, carvacrol and thymol, and remained unchanged over a storage period of three months. The vesicles also retained the antioxidant activity of the essential oil or even amplified it (in the case of antiradical power), and the viability of the skin cells remained unaffected. Hence, the nanoformulation of *S. thymbra* essential oil provides an alternative strategy for its safe and effective use on the skin. Additional in vitro studies are in progress to assess the bioactivity, intracellular targets, and advantages of nanoformulated *S. thymbra* essential oil over application of the free essential oil, and in vivo treatment approaches should be investigated to corroborate in vitro results. Further research validating therapeutic achievements might significantly move the plant-based nanomedicine field forward.

## Figures and Tables

**Table 1:** Volatile compounds in *S. thymbra* essential oil: peak number (as reported in Figure 1), retention time (*tr*), retention index (RI), and amount (% peak area ± SD; *n* = 3).

Peak No.	*tr* (min)	RI	Compound	% ± SD
1	6.17	923	α-Thujene	3.41 ± 0.15
2	6.40	930	α-Pinene	4.38 ± 0.19
3	6.95	946	Camphene	1.30 ± 0.06
4	7.97	974	Sabinene	0.61 ± 0.02
5	8.06	976	β-Pinene	2.46 ± 0.09
6	8.24	980	1-Octen-3-ol	0.58 ± 0.02
7	8.55	988	3-Octanone	0.09 ± 0.00
8	8.74	992	β-Myrcene	2.84 ± 0.01
9	8.92	996	3-Octanol	0.24 ± 0.01
10	9.23	1003	α-Phellandrene	0.59 ± 0.03
11	9.47	1010	3-Carene	0.12 ± 0.01
12	9.76	1017	α-Terpinene	3.76 ± 0.16
13	10.12	1026	*p*-Cymene	7.27 ± 0.03
14	10.28	1030	Limonene	1.09 ± 0.04
15	10.76	1041	(Z)-β-Ocimene	1.54 ± 0.07
16	11.09	1049	*o*-Cymene	1.45 ± 0.06
17	11.21	1051	(E)-β-Ocimene	1.50 ± 0.07
18	11.68	1061	γ-Terpinene	20.38 ± 1.05
19	11.95	1067	cis-Sabinene hydrato	0.21 ± 0.01
20	12.92	1086	Terpinolene	0.12 ± 0.01
21	12.96	1087	*p*-Cimenene	0.04 ± 0.00
22	13.33	1094	trans-Sabinene hydrate	0.07 ± 0.00
23	13.49	1097	Linalool	0.13 ± 0.01
24	16.37	1164	Borneol	3.71 ± 0.17
25	16.93	1176	Terpinen-4-ol	0.40 ± 0.01
26	17.57	1190	Terpineol	0.09 ± 0.00
27	20.03	1246	Thymol methyl ether	6.07 ± 0.26
28	20.27	1251	α-Thymoquinone	0.20 ± 0.01
29	22.35	1296	Thymol	23.88 ± 1.22
30	22.64	1302	Carvacrol	7.08 ± 0.31
31	24.88	1361	Thymol acetate	0.45 ± 0.02
32	25.72	1381	Copaene	0.02 ± 0.00
33	26.08	1390	β-Bourbonene	0.02 ± 0.00
34	27.53	1424	β-Caryophyllene	2.59 ± 0.12
35	28.91	1456	α-Humulene	0.35 ± 0.01
36	29.90	1478	γ-Muurolene	0.04 ± 0.00
37	30.04	1481	Germacrene D	0.26 ± 0.01
38	31.78	1523	δ-Cadinene	0.05 ± 0.00
39	34.00	1580	Caryophyllene oxide	0.32 ± 0.01
			Total identified	99.73 ± 4.63
			Not identified	0.27 ± 0.01

**Table 2:** Mean diameter (MD), polydispersity index (PI), and zeta potential (ZP) of liposomes and Et-PEVs. Values are the means ± SD (*n* > 10). ***: *p* < 0.001 vs. empty liposomes; °°°: *p* < 0.001 vs. empty Et-PEVs; ^§§§^ and ^§§^: *p* < 0.001 and 0.01, respectively, vs. *S. thymbra* liposomes. The entrapment efficiency (EE) of two monoterpenoid phenols, carvacrol and thymol, of *S. thymbra* essential oil loaded in the vesicles is also reported. Values are the means ± standard deviations (SD; *n* = 4).

	MD (nm ± SD)	PI ± SD	ZP (mV ± SD)	EE (% ± SD)
Empty liposomes	96 ± 3.8	0.33 ± 0.04	−12 ± 1.9	--
*S. thymbra* liposomes	*** 86 ± 0.2	*** 0.20 ± 0.04	*** −18 ± 3.0	87 ± 9.2 carvacrol 90 ± 8.7 thymol
Empty Et-PEVs	*** 107± 6.9	0.37 ± 0.06	−12 ± 1.8	--
*S. thymbra* Et-PEVs	°°°^§§§^ 79 ± 2.7	°°°^§§^ 0.24 ± 0.03	°°° −17 ± 2.7	95 ± 6.8 carvacrol 97 ± 5.0 thymol

**Table 3:** Fitting parameters and derived parameters (± estimated error from the fit) for SAXS curves of *S. thymbra* liposomes, empty liposomes, *S. thymbra* Et-PEVs, and empty Et-PEVs. *χ*^2^: reduced chi squared, *d*: repetition distance, *η*_1_: Caillé parameter, *N_c_*: number of correlated bilayers; *σ_H_*: polar head Gaussian amplitude, *ρ_H_*: headgroup contrast electron density; *Z_H_*: polar head Gaussian center, *σ_C_*: methyl Gaussian amplitude.

SAXS Parameters	Empty Liposomes	*S. thymbra* Liposomes	Empty Et-PEVs	*S. thymbra* Et-PEVs
*χ* ^2^	1.7	1.8	1.6	1.3
*d* (Å)	58.0 ± 0.4	-	58.3 ± 0.4	61.1 ± 0.9
*η*_1_	0.18 ± 0.04	-	0.09 ± 0.03	0.13 ± 0.08
*N_c_*	3.5 ± 0.4	-	3.7 ± 0.3	3.2 ± 0.8
% correlated bilayers	14 ± 3	-	12 ± 1	6 ± 1
*σ_H_* (Å)	2.94 ± 0.14	3.27 ± 0.2	3.14 ± 0.16	3.10 ± 0.16
*ρ_H_* (e/nm^3^)	123 ± 6	119 ± 6	130 ± 7	122 ± 6
*Z_H_* (Å)	15.1 ± 0.2	15.3 ± 0.2	14.8 ± 0.1	15.3 ± 0.2
σ_C_ (Å)	4.6 ± 1.0	6.0 ± 1.0	6.0 ± 0.9	5.5 ± 0.9

**Table 4:** DPPH^•^ assay data are reported as AA (%) and TE (μg Trolox equivalents/mL solution). Values are the means ± SD of at least three independent experiments, each run in triplicate. *, **: *p* < 0.01 and *p* < 0.001, respectively, vs. *S. thymbra* solution. FRAP assay data are reported as FE (mg Fe^2+^ equivalents/mL solution). Values are the means ± SD of at least three independent experiments, each run in triplicate.

	DPPH^•^ Assay		FRAP Assay
	AA (%)	TE (µg Trolox Equivalents/mL)	FE (mg Fe^2+^ Equivalents/mL)
*S. thymbra* solution	65 ± 1.9	124 ± 6.1	12 ± 0.22
*S. thymbra* liposomes	** 84 ± 2.3	** 164 ± 4.2	12 ± 1.12
*S. thymbra* Et-PEVs	* 85 ± 5.0	* 171 ± 11.0	12 ± 1.46
Empty liposomes	60 ± 3.5	116 ± 2.0	1.2 ± 0.22
Empty Et-PEVs	69 ± 9.8	142 ± 9.5	1.2 ± 0.21

## Data Availability

The analyzed data sets generated during the present study are available from the corresponding author upon reasonable request.
